# How recreational therapies impact physiological and psychosocial outcomes in cancer patients: a review

**DOI:** 10.3389/or.2026.1726683

**Published:** 2026-03-13

**Authors:** Marika D’Oria, Calogero Casà, Cristina Cenci, Domenico Fusco, Beatrice Di Capua, Edoardo Vergani, Laura Monti, Anna Cardillo, Francesco Miccichè, Emilio Bria, Luca Tagliaferri, Maria Antonietta Gambacorta, Giampaolo Tortora, Vincenzo Valentini

**Affiliations:** 1 Center of Excellence in Oncology, Ospedale Isola Tiberina – Gemelli Isola, Rome, Italy; 2 Radiotherapy Unit, Center of Excellence in Oncology, Ospedale Isola Tiberina – Gemelli Isola, Rome, Italy; 3 Digital Narrative Medicine (DNM s.r.l.), Rome, Italy; 4 Emergency Unity, Urgency Medicine and Internal Medicine, Ospedale Isola Tiberina – Gemelli Isola, Rome, Italy; 5 Psychoncology Unit, Center of Excellence in Oncology, Ospedale Isola Tiberina – Gemelli Isola, Rome, Italy; 6 Medical Oncology Unit, Center of Excellence in Oncology, Ospedale Isola Tiberina – Gemelli Isola, Rome, Italy; 7 Department of Medicine and Translational Surgery, Faculty of Medicine and Surgery, Università Cattolica del Sacro Cuore, Rome, Italy; 8 Oncological Radiotherapy Unit, Department of Diagnostic Imaging and Oncological Radiotherapy, Fondazione Policlinico Universitario A. Gemelli IRCCS, Rome, Italy; 9 Department of Radiological and Hematological Sciences, Faculty of Medicine and Surgery, Università Cattolica del Sacro Cuore, Rome, Italy; 10 Comprehensive Cancer Center, Fondazione Policlinico Universitario A. Gemelli IRCCS, Rome, Italy

**Keywords:** integrative care, oncology, physiological biomarker, psychosocial outcome, recreational therapy

## Abstract

Recreational Therapies (RecT) (e.g., art, dance, music, yoga, aromatherapy, Virtual Reality) are non-invasive interventions capable of enhancing the biopsychosocial wellbeing in patients, targeted to regenerate the existential dimensions of illness experience. While widely appreciated for their positive impact on quality of life, the specific biological and psychological mechanisms through which RecT exert their benefits remain underexplored in oncology. This review maps and critically discusses current evidence on the clinical impact of RecT across various stages of cancer and types of interventions, with a particular focus on targeted outcomes such as cortisol modulation, heart rate regulation, immune response, depression, anxiety, coping skills, and social support. Moreover, the review highlights how RecT may contribute to the mitigation of treatment-related side effects, including nausea, fatigue, and sleep disturbances. By synthesizing recent findings, we provide a comprehensive framework for understanding the role of RecT as integrated, evidence-informed components for oncology rehabilitation during and after therapy. This work aims to support the design of more personalized and effective supportive care strategies that resonate with patients’ values and enhance treatment adherence, resilience, and overall health.

## 1 Introduction

Cancer patients experience several challenges during their therapeutic journey, such as physical (e.g., pain, fatigue, stress) (1), psychosocial (e.g., anxiety, depression, self-esteem, sexuality), and organizational (length of hospital stay, care fragmentation, financial toxicity), and spiritual (2, 3). The World Health Organization highlighted several benefits of integrative interventions for oncology patients in disease prevention, management, and treatment, especially physical and psychosocial (4). Besides, Social Prescribing is a practice that allows clinicians prescribe artistic and cultural activities to patients (5), in order to rehabilitate one’s physical, psychological, social, and spiritual dimensions that cancer can disrupt and fragment, as a result of a traumatic experience (6).

In this scenario, cancer patients can reconnect with several dimensions of their health that have been disrupted by a life-changing diagnosis, while they are sustained in creating new meanings that can be regenerative of their perception of their own human dimension, going beyond simply improving biopsychosocial wellbeing and embracing relational, symbolic, and transcendent dimensions that profoundly enrich the course of treatment (6); therefore, the recreational purpose of such activities goes beyond leisure and distraction.

Recreational Therapies (RecT) are a person-centered approach that uses planned, purposeful recreation as interventions to improve and maximize an individual’s physical, social, emotional, and spiritual wellbeing and overall quality of life (QoL) (7). RecT also include leisure activities as a therapeutic tool to help individuals improve their clinical parameters, regain independence, enhance functional abilities, develop coping skills, and achieve holistic health outcomes. Certified specialists design and implement individualized treatment plans based on a patient’s interests, strengths, and assessed needs; these plans often include activities such as arts, music, dance, animal interactions, storytelling, and community activities (7, 8). RecT is applied in several settings including hospitals, rehabilitation centers, mental health facilities, nursing homes, community centers, and recovery programs; it supports rehabilitation and reintegration of patients by providing meaningful engagement, reducing stress, improving mood, enhancing social connections, and fostering skills that transfer to daily life (9).

In this review, the term RecT is therefore used as an umbrella construct referring to structured, non-pharmacological interventions that activate experiential, expressive, relational, or immersive processes with therapeutic intent. RecT are not defined by a single discipline or therapeutic school, nor are they intended to replace medical or psychological treatments, but rather to function as complementary and supportive interventions within integrative oncology. Despite their heterogeneity, RecT are considered together in this review because they share a common functional core: they activate patient engagement through lived experience, modulating stress-related, emotional, relational, and meaning-making processes that are relevant across physiological and psychosocial domains.

This review aims at mapping what RecT activities can have a positive impact on physiological and psychosocial parameters, as well as to highlight the evidence of using RecT for specific diseases, therapies, and stage of cancer, to foster integrative care in oncology.

## 2 Methods

This review was conducted as a structured narrative review with a systematic search and mapping approach, aimed at synthesizing current evidence on the physiological and psychosocial impact of RecT in oncology. The review protocol was registered on PROSPERO (ID: CRD420250637060) to ensure transparency in the search strategy, eligibility criteria, and analytical approach. While the review does not aim to perform a quantitative synthesis or meta-analysis, protocol registration was adopted to reduce selection bias and to document methodological decisions *a priori*, in line with current recommendations for transparent evidence synthesis in integrative and supportive oncology.

### 2.1 Search strategy

A literature search was conducted in PubMed to identify peer-reviewed studies published between January 2020 and May 2025, to capture recent evidence in integrative and supportive cancer care. The search included randomized controlled trials, non-randomized clinical studies, pilot studies, and relevant meta-analyses.

Articles published in English were identified by using multiple thematic search strings, combining oncology-related terms (oncology, cancer patient, oncology patient) with specific forms of interventions including: aromatherapy; art therapy; art making; cinematherapy; dance; dance therapy; forest therapy; forest bathing; *shinrin-yoku*; laughter; laughter therapy; museum; theater; concert; meditation; music; music therapy; pet therapy; animal-assisted therapy; bibliotherapy; singing; choir; social robots; chatbots; conversational agents; spirituality-related practices (e.g., spiritual, prayer); virtual reality; narrative medicine; digital narrative medicine; expressive writing; yoga.

A total of 18 distinct search queries were performed, each combining intervention-specific terms with oncology-related keywords in the title or abstract. This approach was adopted to improve sensitivity and ensure adequate coverage of heterogeneous interventions that may be indexed under different terminologies. All records retrieved through the different search strings were merged, duplicates were removed, and titles, abstracts, and full texts were screened according to predefined inclusion and exclusion criteria.

### 2.2 Study selection and synthesis

Studies were included if they:

- Involved adult cancer patients, at any disease stage.
- Investigated RecT as non-pharmacological and non-psychotherapy interventions.
- Reported physiological outcomes (e.g., biomarkers, functional parameters) and/or psychosocial outcomes (e.g., emotional distress, coping, quality of life).

Studies were excluded if they:

- Focused exclusively on pharmacological or psychotherapy-based interventions.
- Involved healthy populations, caregivers, or healthcare professionals only.
- Were grey literature, editorials, opinion letters, or corrigenda.
- Were out of scope from inclusion criteria.
- Used the term “ART” to indicate Advanced Radiation Therapy rather than recreational or artistic practices.

Eligibility assessment was performed by two independent reviewers, with disagreements resolved through discussion. Articles full-text assessment completed by August 2025. During full-text assessment, backward and forward citation tracking (snowballing method) was conducted to identify additional relevant publications not retrieved through the initial PubMed search. Studies identified through this process were evaluated using the same inclusion and exclusion criteria applied to database-derived records. This approach resulted in the inclusion of (N = 3) qualitative studies, which were retained to enrich interpretative synthesis, especially in areas where quantitative evidence remains limited.

### 2.3 Data analysis

Due to the heterogeneity of interventions in outcome measures, RecT application, sampling, and study designs, quantitative synthesis was not feasible. Therefore, findings were synthesized through a narrative and analytical approach, describing RecT potential impact according to:

1. Physiological parameters.
2. Psychosocial parameters.
3. Oncological condition.

**FIGURE 1:**
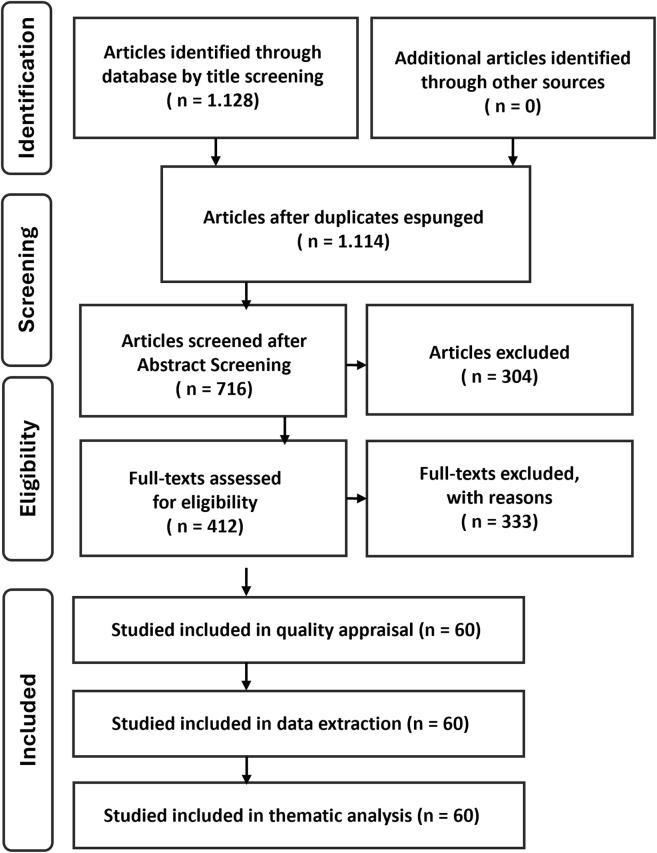
Study selection flowchart.

A PRISMA-ScR–inspired flowchart is provided to document transparency of the process, not to imply systematic exhaustiveness (Figure 1). Interpretation of results and conclusions were discussed and agreed upon by all authors. A total of N = 60 articles were included in this narrative review (Table 1).

**TABLE 1:** Overview of Recreational Therapies (RecT) and main reported outcomes in oncology.

Recreational therapies	Typical population	Physiological oucomes	Psychosocial outcomes	References
Aromatherapy	Adult patients Pediatric patients	↓ Fatigue ↓ Nausea ↓ Vomiting ↑ Sleep quality ↑ Quality of life	↓ Anxiety ↓ Depression ↑ Daily functioning ↑ Emotional wellbeing	(57, 58, 90, 94)
Art-making (e.g., painting)	Adult patients Pediatric patients	​	↓ Anxiety ↓ Depression ↑ Emotional wellbeing ↑ Hope ↑ Quality of life ↑ Social support	(59–62)
Bibliotherapy (books, poetry)	Adult patients Pediatric patients Leukemia Osteosarcoma	↓ Pain ↑ Quality of life	↓ Anxiety ↓ Depression ↓ Distress ↑ Emotional wellbeing ↑ Hope	(40, 84)
Cinematherapy, video	Adult patients Pediatric patients Gynecological cancer	↓ Fatigue ↓ Pain ↑ Quality of life	↓ Anxiety ↑ Emotional functioning	(45, 46, 79, 85)
Conversational agents (chatbot, robots)	Adult patients Pediatric patients Breast cancer Gastrointestinal cancer	↓ Pain	↓ Distress ↑ Adherence ↑ Engagement ↑ Satisfaction	(41, 78, 81, 82)
Dance and movement	Adult patients Breast cancer Cancer survivors	↓ Fatigue ↑ Body motion ↑ Exercise capacity ↑ Physical functioning ↑ Quality of life	↓ Anxiety ↓ Depression ↓ Mood disturbances ↑ Couple trust ↑ Body image perception ↑ Self-esteem ↑ Social support	(10, 11, 33–36, 38, 63)
Laughter	Adult patients Lung cancer Terminally ill patients	↓ Fatigue ↓ Pain ↑ Exercise capacity ↑ Physical functioning	↓ Anxiety ↓ Depression ↓ Distress ↓ Mood disturbances ↑ Compliance ↑ Resilience	(37, 39, 42–44)
Live art in hospital (museum, concerts, paintings)	Adult patients Lymphoma Gynecological cancer	↓ Blood pressure ↓ Cortisol ↓ Heart rate ↑ Quality of life	↓ Anxiety ↓ Depression ↓ Distress ↑ Coping ↑ Emotional support ↑ Social interaction	(14, 64, 65, 73, 74, 83)
Music	Adult patients Breast cancer Gynecological cancer Cancer survivors	↓ Blood pressure ↓ Heart rate ↓ Fatigue ↓ Pain ↓ Respiratory rate ↑ Quality of life	↓ Anxiety ↓ Depression	(12, 13, 19, 21, 31, 48, 86)
Nature	Adult patients Breast cancer Cancer survivors	↓ Cortisol ↓ Fatigue ↑ Immune NK cell ↑ Perforin ↑ Granzyme B ↑ Serotonin	↓ Depression ↓ Distress	(22, 32)
Pet therapy	Adult patients Pediatric patients Brain tumor Leukemia	↓ Blood pressure ↓ Fatigue ↓ Irritation ↓ Heart rate ↓ Pain ↑ Appetite ↑ Quality of life	↓ Anxiety ↓ Depression ↓ Distress ↑ Self-perceived health ↑ Sense of coherence ↑ Socialization	(15, 16, 47, 52, 66)
Singing	Adult patients Lung cancer Advanced stage cancer Cancer survivors	↓ Pain	↓ Anxiety ↓ Depression ↓ Distress ↑ Emotional wellbeing ↑ Empowerment ↑ Self-esteem ↑ Sense of purpose ↑ Social support	(51, 67, 68)
Spirituality (prayers, mindfulness)	Adult patients Pediatric patients Breast cancer Gynecological cancer Prostate cancer Glioblastoma Advanced cancer	↓ Blood pressure ↓ Cortisol ↓ Fatigue ↓ Inflammation ↓ Pain ↑ Immune system ↑ Overall survival	↑ Adherence ↑ Engagement ↑ Satisfaction	(17, 18, 23, 30, 49, 54, 70–72, 80)
Virtual Reality	Adult patients Acute leukemia	↓ Fatigue ↓ Pain ↑ Muscular movements	↓ Anxiety ↓ Depression ↓ Distress ↑ Satisfaction	(50, 75)
Writing (expressive writing, narrative medicine)	Adult patients Breast cancer Metastatic prostate cancer Bone and soft tissue sarcoma Cancer survivors	↑ Quality of life	↑ Coping ↑ Disease awareness	(76, 77, 87)
Yoga	Adult patients Breast cancer Early cancer Cancer survivors	↓ Cortisol ↓ Fatigue ↓ Heart rate ↓ Inflammation ↓ Nausea and vomiting ↑ Immune function ↑ Quality of life ↑ Sleep quality	↓ Anxiety ↓ Depression ↓ Distress ↓ Mood disturbances	(24, 25, 55, 69, 88)

The final number of included studies (N = 60) reflects a process of conceptual and thematic saturation rather than a fixed numerical threshold. The literature search was conducted through 18 distinct intervention-focused queries, each designed to capture heterogeneous forms of RecT that are often indexed under different terminologies.

Results from these searches were merged, duplicates were removed, and studies were selected based on their relevance to the review’s conceptual framework and targeted outcomes. In addition, backward and forward citation tracking (snowballing) was employed to identify key qualitative and interdisciplinary studies (N = 3) that were not consistently retrievable through database indexing alone. This approach is consistent with methodological standards for structured narrative reviews in heterogeneous and emerging fields.

## 3 Physiological parameters

**FIGURE 2:**
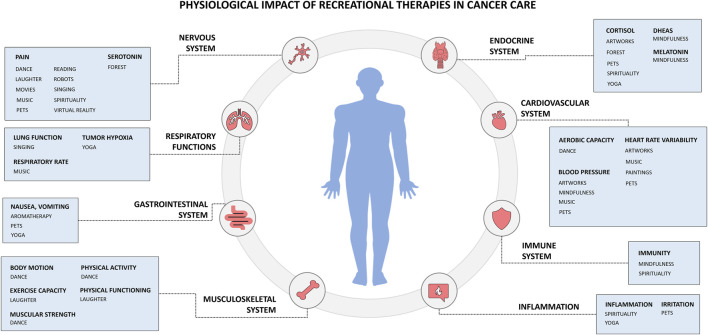
Physiological impact of Recreational Therapies (RecT) in cancer care.

Emerging evidence suggests RecT ameliorate cancer patients’ physiological parameters, impacting multiple systems, biomarkers, and physical functions (Figure 2).

### 3.1 Cardiovascular system

Several RecT were associated with cardiovascular-related outcomes, particularly aerobic capacity, blood pressure, and heart rate variability (Figure 2). Movement and dance-based interventions showed consistent benefits on aerobic capacity and overall functional fitness (10, 11), supporting cardiovascular endurance in cancer patients engaged in structured or community-based activities.

Blood pressure modulation emerged across different RecT, with music listening (12, 13), visual art exposure (14), pet therapy (15, 16), and mindfulness-based practices showing favorable effects (17, 18). These interventions appear to act through stress reduction and autonomic regulation, although evidence remains heterogeneous and context-dependent. Notably, studies investigating live music (19) during chemotherapy suggest potential benefits, but findings are still preliminary and warrant further investigation.

Heart rate variability, a marker of (autonomic nervous system activity and overall cardiovascular health (20), was positively influenced by selected RecT, particularly music (21), visual art observation (14), and pet therapy (15). Taken together, these findings indicate that RecT may contribute to cardiovascular regulation primarily through stress-related and autonomic pathways, complementing conventional oncological care.

### 3.2 Endocrine system

Several RecT were associated with endocrine-related outcomes, particularly biomarkers involved in stress regulation and circadian rhythms. Across studies, cortisol emerged as the most frequently assessed marker of stress response. Consistent reduction of cortisol levels were reported in cancer patients exposed to forest therapy (22), visual art observation (14), pet therapy (16), mindfulness (18, 23) and yoga (24, 25). Given the established association between elevated cortisol levels, poor oncological prognosis, and reduced tumor sensitivity to chemotherapy, these findings are of particular interest in supportive cancer care. However, cortisol assessment in oncological settings remains methodologically challenging, due to the frequent use of corticosteroid during treatment, prompting interest in complementary biomarkers of linked to adrenergic activity (26).

Dehydroepiandrosterone sulfate (DHEA-S), a biomarker for adrenal gland function and immune modulation, was less frequently investigated but showed sensitivity to mind-body interventions like mindfulness. Alterations in DHEA-S have been associated with fatigue, depression, and poor prognosis after chemotherapy (27, 28), suggesting a potential role of mindfulness in modulating neuroendocrine balance (17).

Circadian regulation, assessed through melatonin levels (29), also emerged as a relevant endocrine parameter. Mindfulness-based interventions (17) were associated with improvements in melatonin secretion, supporting sleep-wake regulation in cancer patients. Given the relationship between circadian disruption and tumor aggressiveness in selected cancers, these findings suggest that mindfulness may contribute to endocrine homeostasis through stress reduction and circadian stabilization mechanism.

### 3.3 Immune system

Evidence regarding the impact of RecT on immune-related outcomes remain limited and heterogeneous. A small number of studies explored immune biomarkers in cancer patients exposed to spiritual practices like mindfulness (18, 30), with preliminary findings suggesting potential modulation through stress-related and mind-body pathways. Effects of music (31) and nature-based interventions (22) were explored less consistently.

Cytotoxic immune markers, including granzyme B (GrB) and perforin, (both involved in antitumor immune responses and immunotherapy sensitivity) were assessed in a limited number of studies. Forest therapy intervention were explored in relation to these biomarkers; however, findings did not demonstrate clinically meaningful variations (32).

Overall, current evidence does not support robust conclusions regarding direct immune modulation by RecT. Rather, immune-related effects appear indirect and may be mediated through neuroendocrine and stress-regulatory pathways, as suggested by more consistent findings in endocrine and autonomic domains. These results highlight the need for more targeted and methodologically robust studies to clarify whether and how RecT may influence immune function in oncology.

### 3.4 Musculoskeletal system

Across musculoskeletal outcomes, body motion-based interventions emerged as particularly relevant in oncology, both as physical activity and as facilitators of functional engagement during treatment. Dance and movement-based therapies were consistently associated with improvements in motor-related outcomes, including mobility and functional capacity, across different cancer populations (33–36).

Exercise capacity, a key indicator of physical resilience and treatment tolerance, showed improvements in patients exposed to selected RecT, with preliminary evidence suggesting a beneficial role of laughter-based activities (37). Similarly, muscular strength, an outcome closely linked to, prognosis and vulnerability to treatment complications, appeared to benefit from dance-based and movement-oriented RecT functioned not only as therapeutic modalities but also as accessible entry point to sustained physical engagement (10).

Importantly, several interventions contributed to the introduction or maintenance of regular physical activity, a factor known to support long-term outcomes in cancer survivors. In this context, dance (38) and laughter yoga (37, 39), functioned not only as therapeutic modality but also as accessible entry point to sustain physical functioning.

Taken together, these findings suggest that movement and body-centered RecT preferentially impact functional and musculoskeletal domains, supporting physical functioning and autonomy during and after cancer treatment.

### 3.5 Nervous system

Nervous system-related outcomes concern pain modulation and, to a lesser extent, neurochemical markers. Across RecT interventions, outcomes converged on reducing pain in patients undergoing cancer treatments, including those experiencing treatment-related or neuropathic pain. A wide range of RecT were associated with pain relief, spanning expressive, relational, and immersive modalities. These poetry (40), dance (34), interaction with robots (41), laughter therapy (42–44), movies (45, 46), pet therapy (47), music (48), spiritual interventions (49), and Virtual Reality (VR) (50). Despite heterogeneity in study design and outcome measures, findings consistently suggest that RecT may contribute to pain modulation through attentional, emotional, and sensory mechanisms.

Neurochemical outcomes were less frequently investigated. Serotonin-related measurements were explored in a limited number of studies, with forest-based interventions showing potential association with reduced serotonin levels (22). Given the lack of more evidence, conclusions regarding direct neurochemical modulation by RecT remains preliminary.

### 3.6 Respiratory function

Respiratory-related outcomes by RecT were explored in a limited number of studies and included lung function parameters, tumor hypoxia, and respiratory rate. Overall, evidence in this domain remains heterogeneous.

Lung function, primarily investigated in patients with lung cancer, showed no significant improvement following singing-based interventions (51), suggesting that while such activities may offer psychosocial or expressive benefits, their impact on objective pulmonary function remains limited.

Tumor hypoxia, a biomarker associated with aggressive malignancy and poor prognosis, was assessed in only a few studies. Music listening (12) and pet therapy (52) did not demonstrate significant effects for this aspect, whereas yoga-based practices (24) show more consistent, although still preliminary, associations with hypoxia-related parameters.

Respiratory rate (53), an early indicator of physiological deterioration, was more responsive to selected RecT. Music-based interventions (12) were associated with improvements in respiratory rate, likely reflecting autonomic regulation and relaxation-related mechanisms rather than direct pulmonary effects.

Current findings suggest that RecT may influence respiratory-related outcomes indirectly (through stress reduction and autonomic modulation) rather than through direct changes in pulmonary function, highlighting the need for more targeted research in this area.

### 3.7 Inflammation

Inflammation-related outcomes were explored with patients practicing individual prayers (54) and yoga (55) and suggesting a potential role in modulating such pathways. Treatment-related inflammatory manifestation, including irritation phenomena such as mucositis and dermatitis, were also addressed. In this context, pet therapy (47) showed favorable effects in reducing irritation-related symptoms, pointing towards an indirect influence of RecT on inflammatory burden through stress reduction, comfort, and supportive care mechanisms.

While evidence remains preliminary, these findings suggest that specific RecT may contribute to inflammation modulation primarily through neuroendocrine and psychosocial pathways, rather than direct anti-inflammatory effects.

### 3.8 Gastrointestinal system

Gastrointestinal symptoms, including nausea, vomiting, and appetite loss, were among the most consistently addressed treatment-related outcomes in the reviewed literature (56). Across studies, aromatherapy (57, 58) emerged as the intervention with the strongest evidence in reducing such symptoms, supporting its role as a complementary strategy in oncology. Additional RecT, including animal-assisted interactions (15) and yoga (24), were also associated with improvements in gastrointestinal symptoms, although evidence was more heterogeneous. By contrast studies investigating the effects of live music during treatment did not demonstrate consistent benefits for these symptoms (19).

These findings suggest that aromatherapy may effectively alleviate gastrointestinal side effects of cancer treatment, potentially supporting treatment adherence and quality of life, while highlighting the need for further comparative research in this area.

## 4 Psychosocial parameters

**FIGURE 3:**
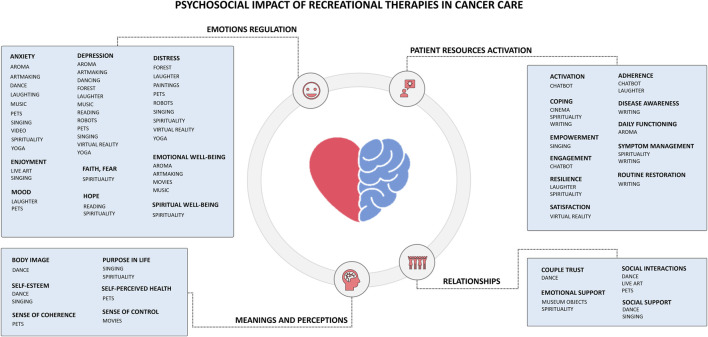
Psychosocial impact of Recreational Therapies (RecT) in cancer care.

Evidence suggests RecT can ameliorate cancer patients’ psychosocial parameters (e.g., anxiety, depression, social support), reduce their emotional distress, and facilitate adherence to treatment (Figure 3). Despite psychotherapy-based and pharmacological-based therapy have been widely proven to be beneficial to this extent, in this article we are considering such aspects as inevitably underlying and part of the oncologic journey, by investigating them through the lenses of the RecT.

### 4.1 Emotions regulation

Emotion regulation-related outcomes were among the most consistently investigated psychosocial domains across RecT. Reduction in anxiety, depressive symptoms, and psychological distress emerged as convergent findings in patients exposed to a wide range of RecT, spanning sensory, expressive, relational, and immersive modalities.

Anxiety and depressive symptoms were particularly responsive to multiple interventions, including aromatherapy (58), art-based and expressive activities (e.g., art-making (59–62), poetry reading (40), dance (63)), music-based interventions (both recorded (12, 13) and live (64, 64)), animal-assisted interactions (52, 66), singing (67, 68), yoga (69), VR,^50^spirituality-related practices (70–72), and art appreciation in hospital environments (73, 74). Despite heterogeneity in outcome measurements and study design, the consistency of these findings suggest a robust role of RecT in supporting emotional regulation during cancer treatment.

Psychological distress (a multidimensional construct reflecting emotional overload and stress reactivity) was also reduced across different RecT. Interventions emphasizing social connection (e.g., laughter-based activities (37, 39), pet therapy (47), group singing (67)), immersive engagement (social robots (41), VR (75), nature-based experiences (22)), and mind-body practices showed particularly favorable effects, highlighting the importance of relational and experiential dimensions in distress modulation.

Beyond symptom reduction, several RecT contributed to positive emotional states and resources. Improvements in emotional wellbeing, (5, 25), faith (70), fear of cancer recurrence (71), and spiritual wellbeing (49) were reported across studies involving art engagement (60), music (48), live performance (65), singing (68), prayer (72), and spiritual practices (70). These outcomes point toward a broader role of RecT in fostering meaning-making, positive affect, and adaptive coping, rather than merely alleviating negative emotional states.

These findings indicate that RecT support emotion regulation in cancer patients through multiple complementary pathways, including attentional engagement, emotional expression, social connection, and existential meaning. This multidimensional impact underscores their relevance within integrative and supportive cancer care, particularly for addressing the emotional and existential burden with illness and treatment.

### 4.2 Meanings and perceptions

Beyond emotional symptom modulation, several RecT were associated with changing patients’ meanings, perceptions, and self-related constructs. These outcomes reflect how individuals interpret their bodies, health, and life trajectory in the context of cancer.

Body-related perceptions emerged as a relevant domain, particularly in relation to interventions involving movement and embodied expression. Dance-based therapies (34, 63) were associated with improvements in body image, supporting patients in renegotiating their relationship with bodily changes induced by disease and treatment.

Existential dimensions, including purpose in life and meaning in life, were influenced by RecT emphasizing collective participation and spiritual engagement. Singing in choirs and spirituality-related practices were associated with enhanced sense of purpose (49, 67) and meaning, suggesting that shared expressive and reflective activities may support existential coherence and acceptance of illness.

Self-related perceptions, such as self-esteem and self-perceived health, were also responsive to selected RecT; interventions involving social interaction and relational engagement (particularly group singing (67), dance-based activities (63), and animal-assisted interventions (52)) were associated with more positive self-evaluations and subjective health perceptions.

Cognitive-existential constructs related to orientation and agency, including sense of coherence and sense of control, were less frequently investigated but showed preliminary associations with RecT fostering relational safety and environmental engagement. Pet therapy was linked to improvements in sense of coherence (52), while exposure to visual artworks within healthcare settings showed potential to enhance patients’ perceived sense of control (73), although evidence remains limited.

### 4.3 Patient resources activation

Patients can be empowered if they are enabled, engaged, and involved in the therapeutic process, from understanding their disease to making informed decisions, from education promotion to motivation for continuing a therapy. Across studies, RecT contributed to enhancing patients’ awareness, engagement, and sense of agency, facilitating participation in decision-making, self-management, and adaptive coping.

Interventions involving narrative and digital tools, including digital narrative medicine (76, 77) and chatbot-based interactions (78), were associated with increased disease awareness, patient activation, and engagement. These approaches appeared particularly relevant for supporting information processing, health literacy, and sustained interaction with care pathways, potentially contributing to improved adherence and satisfaction.

Collective and expressive RecT, such as group singing (67), cinematherapy (79), laughter yoga (37), and spiritually oriented practices (80), were associated with patient empowerment, resilience, and coping. These interventions supported emotional and motivational resources, enabling patients to mobilize internal strengths and sustain engagement throughout treatment.

Finally, patient experience-related outcomes were positively influenced by technology-mediated RecT. VR interventions (50) and chatbot-supported communication (81, 82) were associated with increased patient satisfaction, highlighting the role of innovative tools in enhancing the perceived quality and personalization of care.

### 4.4 Relationships

Relational outcomes were addressed across several RecT, highlighting the role of interpersonal connections in supporting cancer patients’ wellbeing and engagement in care. Interventions fostering shared experiences and embodied interaction were associated with improvements in relational quality, emotional support, and social connectedness.

Within intimate relationships, dance therapy was associated with enhanced couple trust, suggesting that shared movement and coordinated activity among cancer patients and their partners might strengthen mutual attunement and communication (38). Such relational dynamics are clinically relevant, as supportive partnerships are linked to better treatment adherence, engagement in decision making, and overall wellbeing.

Emotional support by peers emerged as key relational dimension influenced by RecT. Interventions facilitating interpersonal presence (e.g., group-based activities (10, 15, 87), spiritual practices (72, 80), and tactile engagement with cultural objects (73, 83)) were associated with reduced feelings of isolation and enhanced emotional support. These effects underline the importance of shared meaning and relational safety in mitigating the psychological burden.

Broader social interaction and social support were also positively influenced by RecT that encourage collective participation. Dance sessions (34), live artistic experiences within healthcare settings (57) and animal-assisted interventions (15) facilitated social connection and peer support, contributing to improved QoL and adaptive coping.

Overall, these findings suggest that RecTs support relational processes in oncology by creating shared experiential spaces that foster trust, emotional support, and social belonging, reinforcing the interpersonal foundations of integrative and person-centered cancer care.

## 5 Oncological conditions

### 5.1 Type of cancer

**FIGURE 4:**
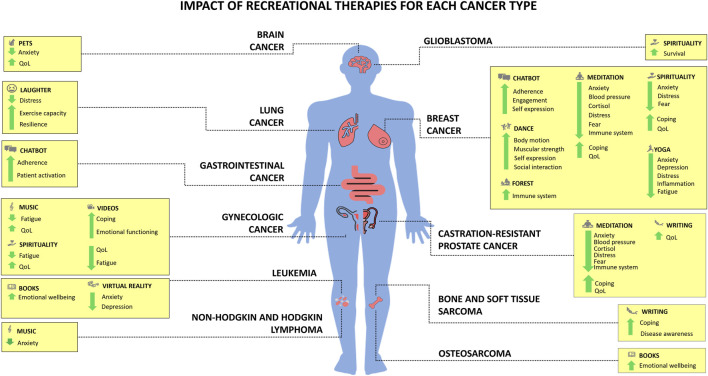
Impact of Recreational Therapies (RecT) for each cancer type.

Evidence on the application of varies across cancer types (Figure 4), reflecting differences in symptom burden, treatment intensity, and psychosocial needs. Rather than indicating tumor-specific efficacy, current findings suggest that RecT tend to address shared clinical and experiential challenges that emerge within specific oncological contexts.

Brain tumors are characterized by high psychological distress, cognitive burden, and limited therapeutic options. In this population, pet therapy with exposure to personal-related imagery of emotions were associated with improvements in QoL as well as anxiety levels (52). Moreover, higher levels of spirituality and resilience were associated with longer overall survival, although these findings derive from observational data and do not support causal inference (80).

In sarcomas and rare tumors, where patients often experience prolonged diagnostic trajectories and high uncertainty, narrative-based interventions appear particularly relevant. Digital narrative medicine facilitated disease awareness and adaptive coping strategies in patients with bone and soft tissue sarcoma (76, 77), while bibliotherapy supported emotional wellbeing among adolescents with osteosarcoma (84). These findings suggest that meaning-oriented RecT may be especially valuable in conditions marked by rarity and existential ambiguity.

Breast cancer represents the most extensively studied population, reflecting both its prevalence and the long-term functional and psychosocial sequelae of treatment. In this group, movement-based RecT such as dance and Dance Movement Therapy consistently improved shoulder mobility, muscular strength, physical capacity, body image, fatigue, and QoL (10, 34, 63). Mind-body practices, including yoga and mindfulness, demonstrated robust effects on anxiety, depression, distress, fatigue, sleep quality, and stress-related biomarkers (e.g., cortisol, inflammatory markers) (55, 71, 88). Nature-based interventions and spiritual practices further contributed to immune and neuroendocrine modulation (17, 18, 22, 30). Digital tools, such as chatbots, supported treatment adherence, patient engagement, and satisfaction (81) by providing a space for expression of unmet informational and emotional needs.

In hematological malignancies, immersive and sensory interventions showed promise. VR reduced anxiety and depressive symptoms in patients with leukemia (75) during intensive treatment phases, bibliotherapy can improve emotional wellbeing (84), while live music (more than recorded music) was associated with anxiety reduction in patients with Hodgkin and Non-Hodgkin lymphoma (19), highlighting the importance of relational and contextual factors in intervention delivery.

Patients with lung cancer, who often experience high symptom burden and functional decline, benefited from laughter yoga (37), which was associated with reduced distress, enhanced resilience, and improved exercise capacity. Similarly, in gastrointestinal cancers, technology-mediated interventions such as chatbot-based communication improved treatment adherence and patient activation (82), supporting self-management during chemotherapy.

In gynecologic cancers, audiovisual and narrative-based RecT, including movie watching (85) and cinematherapy (79), consistently improved emotional functioning, coping,QoL, and cancer-related fatigue. Complementary interventions such as music therapy (86) or spiritual practices (70) further supported fatigue reduction and overall wellbeing. In patients with metastatic castration-resistant prostate cancer, narrative medicine enhanced QoL (87), while mindfulness showed physiological and psychosocial effects comparable to those observed in breast cancer populations (17, 18).

Evidence suggests that RecT effectiveness is less determined by tumor histology *per se* than by the clinical phase, symptom profile, and experiential needs associated with different cancers. This perspective supports the integration of RecT based on patient-centered characteristics.

### 5.2 Stage of the disease

Evidence suggests that the effectiveness of RecT varies across different stages of the oncological trajectory, reflecting changes in symptom burden, psychological needs, and treatment goals over time.

In early stage cancer, particularly in patients with early breast cancer, yoga has been associated with reductions in cortisol levels, anxiety, and depression (88). These findings support the role of RecT in modulating stress-related pathways during active treatment phases, potentially enhancing psychological adjustment and treatment tolerance.

In advanced stage cancer, where symptom control and emotional support become entral therapeutic priorities, relational and spiritually oriented RecT appear particularly relevant. Choral singing performed together with family members has been associated with reductions in pain and anxiety and with improvements in emotional wellbeing (48), highlighting the importance of shared experiential and relational dimensions at this stage. Similarly, individual prayer has been associated with reduced inflammation and anxiety (54), alongside improved symptom management and perceived emotional support.

In terminal stages of illness, RecT focusing on emotional relief and caregiver involvement show promising outcomes. Laughter therapy (39) conducted with family caregivers was associated with reductions in patients’ pain and improvements in mood, while also contributing to lower burnout levels among caregivers, underscoring the dyadic and relational value of such interventions in end-of-life care.

In cancer survivorship, RecT shift toward sustaining long-term health, functional capacity, and resilience. Music-based interventions, whether through listening or active performance, have been associated with improvements in heart rate variability and reductions in cancer-related fatigue (21, 55). Social and movement-based practices, such as dance (34) and choral singing (48) involving caregivers, further support physical activity levels and quality of life. Among RecT, yoga (55), meditation (30), and forest therapy (22) appear particularly effective in modulating neuroendocrine and immune-related biomarkers in survivors, suggesting a role in restoring physiological homeostasis beyond active treatment.

### 5.3 Treatment tolerance

Adverse events from cancer treatments represent a broad and often overlapping spectrum of physical and psychological burdens, ranging from hematological toxicities such as neutropenia, anemia, and thrombocytopenia, to gastrointestinal effects, cardiopulmonary, hepatic, neural, neurological, endocrine and psychological dysfunction. Such toxicities can arise from various cancer therapies and can significantly impact a patient’s QoL as well as treatment outcomes.

Among these adverse events, cancer-related fatigue (89) stands out as one of the most pervasive and distressing, subjective, and persistent symptoms experienced by cancer patients and survivors due to the cancer itself or its treatments. A growing body of evidence suggests that several RecT may help mitigate this symptom, including dance (10, 63) laughter yoga (37, 39), forest therapy (22), watching movies (45), music listening (21), pet therapy (47), and spiritual practices (49).

QoL has therefore emerged as a central outcome in oncology, capturing the multidimensional impact of cancer and its treatments on a patient’s physical, emotional, and social wellbeing. Unlike traditional survival metrics alone, QoL assessment provides crucial insights beyond traditional survival metrics, informing personalized treatment plans, monitoring treatment toxicity, identifying risk factors for progression, and ultimately improving patient-centered care and outcomes. Several forms of RecT have been shown to improve cancer patients’ QoL, including aromatherapy (99), narrative medicine (87), cinematherapy (79) and movies (85), dance (34, 40) and music therapy (12), singing (51), practices like mindfulness (18) and yoga (24).

Beyond subjective outcomes, interest has also grown in understanding if RecT may influence objective healthcare indicators. Length of hospital stay (LOS) (91) is a crucial clinical indicator reflecting the quality of care, resource utilization, and patient outcomes, with prolonged LOS often associated with increased costs, higher risks of complications, and potential negative impacts on patient wellbeing and mortality. While the presence of artworks in the hospital (73) has been investigated as potential RecT, results does not consistently demonstrate a significant reduction in LOS, highlighting the need for more structured and participatory approaches.

At a biological level, oxidative stress (92) plays a pivotal role in cancer initiation, progression, and metastasis through mechanisms involving genomic instability and altered redox signaling. Among RecT, yoga appears particularly promising in modulating oxidative stress markers and restoring redox balance in cancer patients (24), suggesting a possible bridge between experiential practices and molecular outcomes.

Sleep disturbances are another highly prevalent yet often under-recognized concern in oncology. Sleep quality (93) (encompassing sleep onset, continuity, duration, and restorative capacity) is frequently compromised in cancer patients, with consequences as weakened immune function, impaired cognitive and physical performance, increased risk of cancer recurrence, and a diminished QoL. Interventions like aromatherapy (94) with lavender and peppermint essential oils, as well as mindfulness (23) and yoga (88), have been proven to be effective in improving sleep quality among cancer populations.

Survival (95) remains the most traditional endpoint in oncology, typically expressed as the proportion of patients alive at specific time points following diagnosis or treatment initiation. Research shows that higher levels of spirituality and resilience have been associated with improved survival in patients with glioblastoma, underscoring the relevance of existential dimensions in oncological trajectories while calling for cautious interpretation (80).

Finally, treatment time (96) (including the time to diagnosis and treatment initiation, the duration of treatment cycles, and the overall time a patient experiences clinical benefit from therapy), represents a critical temporal dimension in cancer care. These time-related factors directly impact patient outcomes, disease progression, and treatment efficacy. Among RecT, watching audiovisual contents in the treatment room has been shown to reduce treatment time (45), suggesting that experiential interventions may positively modulate both psychological and operational aspects of oncology care.

## 6 Discussion

The physiological benefits of RecT in oncology span multiple body systems and functions, contributing to both treatment tolerance and QoL. Cardiovascular parameters (e.g., heart rate, blood pressure) can improve through music, painting, interaction with pets, and mindfulness; RecT also modulate endocrine biomarkers (e.g., cortisol, DHEA-S, and melatonin) reflecting reductions in stress and circadian dysregulation. Although evidence is still emerging, immune parameters including GrB and perforin have been explored in relation to forest bathing and mindfulness.

Musculoskeletal benefits (e.g., exercise capacity, body functioning) are consistently reported with dance and laughter therapy. The nervous system also responds positively with several RecT, showing analgesic effects and potential modulation of serotonin. In terms of respiratory and gastrointestinal function, yoga and aromatherapy can mitigate treatment-related side effects like nausea and fatigue.

Some RecT can reduce biomarkers of toxicity (e.g., inflammation, oxidative stress) and being spiritual correlates with improved survival; such findings suggest a physiological rationale for incorporating most RecT into integrative cancer care pathways.

RecT have a deep impact on key psychological dimensions in oncology; they alleviate emotional distress (especially depression and anxiety), by offering patients outlets for self-expression and mood regulation; most therapies help reframe patients’ perception of illness, providing meaning-making experiences that foster hope, coping, and resilience. Research investigating spiritual activity (prayers, meditation, and yoga) shows a positive impact on clinical and psychological outcomes, especially stress reduction and sociality enhancement, highlighting a potential strategy that glues together the traumatic disconnection between body and mind.

Group-based therapies (e.g., dance, laughter) enhance peer connection and reduce feelings of loneliness, a known risk factor for poor cancer outcomes; pet therapy and VR also reduce isolation by fostering presence and positive engagement with the environment. RecT can contribute to improved social functioning; however, in studies involving group-based or social interventions, it is often unclear whether the observed benefits is associated to the recreational experience itself or to social interactions. Additionally, the role of activity facilitators is not always clearly described, yet their presence can influence the outcomes of the intervention.

Few research tests the impact of RecT on cancer patients’ therapeutic outcomes and treatment tolerance. Lastly, there is no significant difference in age or gender across cancer patients, who can benefit from RecT more than others.

### 6.1 Limitations of the evidence

One of the main limitations of the current evidence (and consequently of this review) is the substantial heterogeneity characterizing research on RecTs in oncology, which currently precludes meaningful meta-analytic synthesis.

Included studies vary widely in terms of intervention type, patient populations, cancer type and disease stage, outcome measures, and study design. Many involve small samples, pilot designs, or short follow-up periods, limiting generalizability and comparability. Moreover, psychosocial and physiological outcomes are often assessed using heterogeneous tools.

Most studies focus on breast cancer patients, leaving other oncologic populations underrepresented. Involving patients with different tumors may help identify which art forms are most beneficial for specific subgroups. Such interventions should be further investigated for specific cancers and the stage of the disease. This would support better stratification and personalization of interventions. Importantly, this heterogeneity reflects the emerging and interdisciplinary nature of RecT, which are inherently context-sensitive and relational. Future research would benefit from greater methodological convergence, including shared reporting frameworks, clearer characterization of intervention components, and the development of core outcome sets. Such advances would enable more robust quantitative synthesis while preserving the experiential and person-centered dimensions central to RecT.

### 6.2 Future directions

The absence of empirical evidence for certain outcomes in the literature does not necessarily indicate a lack of clinical relevance; rather, it may reflect the fact that specific correlations have not yet been adequately explored or studied. The lack of direct associations should not be misinterpreted as a lack of therapeutic potential.

Several RecT have been studied for specific clinical factors (type of cancer, stage, treatment); however, the response to stimuli (like the stress response and art appreciation) is not universal since it significantly varies on individual sensitivity. Future research could explore which RecT works better according to patient’s preferences.

Research often focuses on cortisol as a biomarker of physiological distress, but such parameter could be affected by cancer treatments. Salivary amylase has been proven to be more accurate in revealing stress levels in cancer patients (98), hence future studies might investigate such value.

Many interventions remain largely absent from current research despite their therapeutic potential. Practices such as pottery, weaving, embroidery, photography, scuba diving, horticulture, and calligraphy offer symbolic-sensorial pathways for emotional processing, identity reconstruction, and rehabilitative outcomes. Future research should explore underutilized forms to expand the palette of creative care in oncology.

It is unclear if, in oncology settings, such activities are always supervised by RecT professionals. Integrative care interventions should be situated within a therapeutic relationship where the health professional deeply understands what RecT best suits for every specific patient can, at specific stages of the disease. Recreational activities should be selected carefully, since sensory stimulation may elicit different effects on people (99). RecT are an expedient used within the clinical pathway for therapeutic purposes, transforming the cancer experience into a holistic transformation that enables the person to become empowered and encourages to talk about the disease to the clinician when words are not enough. In this scenario, research is needed to gain stronger evidence that leads to the prescription of such therapeutic activities in cancer care, related to specific clinical outcomes and the personalized needs of each patient.

Recent SIO–ASCO guidelines provide evidence-based recommendations for integrative approaches primarily in symptom-focused domains (pain, anxiety/depression, fatigue) (97), with stronger support for selected mind–body interventions and specific modalities (e.g., mindfulness-based programs, yoga, music therapy, aromatherapy, and physical exercise in defined contexts). Other RecT remain promising (e.g., nature, movies, writing, spiritual practices) but are supported by heterogeneous evidence and are not yet consistently represented in major guidelines.

RecT are recreational because they re-create a connection between several dimensions of the being (biological, physiological, clinical, relational, societal, and psychological). For instance, such practices might be progressively included “by design” into a regenerative, therapeutic relationship (6) where a new meaning can be created for the whole illness experience for a better quality of existence.
